# Supplementary material for: MetaRibo-Seq measures translation in microbiomes
Source: Nat Commun. 2020 Jun 29;11:3268. doi: 10.1038/s41467-020-17081-z (PMC7324362; doi:10.1038/s41467-020-17081-z)
Supplement: Supplementary file 10 — Supplementary Data 7 [file 41467_2020_17081_MOESM10_ESM.zip › File2/Confidence_High_Taxonomy/58644_out.krona.html]

Javascript must be enabled to view this page.

members
magnitude
magnitudeUnassigned
count
unassigned
taxon
rank

58644\_out

29

29
superkingdom
2

1239
phylum
29

186801
class
29

186802
order
29

29
family
186803

841
genus
29

301301

SRS142781\_contig\_number\_4430
1
species

166486

SRS013158\_contig\_number\_contig-100\_199.42705SRS014287\_contig\_number\_17092SRS014855\_contig\_number\_2135SRS015065\_contig\_number\_16998SRS015579\_contig\_number\_21170SRS016335\_contig\_number\_45583SRS019397\_contig\_number\_12634SRS022137\_contig\_number\_19891SRS024549\_contig\_number\_4303SRS045739\_contig\_number\_contig-100\_18190.52946SRS052027\_contig\_number\_16917SRS054956\_contig\_number\_3208SRS055017\_contig\_number\_27116SRS056273\_contig\_number\_7257SRS056519\_contig\_number\_18525SRS075716\_contig\_number\_4322SRS077454\_contig\_number\_17120SRS098827\_contig\_number\_12604SRS1041038\_contig\_number\_contig-100\_14110.87667SRS104485\_contig\_number\_271SRS142599\_contig\_number\_6455SRS142781\_contig\_number\_11864SRS143070\_contig\_number\_contig-100\_1462.111283SRS143780\_contig\_number\_contig-100\_1811.103683SRS144362\_contig\_number\_44202SRS148159\_contig\_number\_21404SRS148424\_contig\_number\_16068SRS893378\_contig\_number\_17011
species
28
